# Feeding of faba beans (*Vicia faba* L.) enhances the growth performance of lambs

**DOI:** 10.14202/vetworld.2022.906-910

**Published:** 2022-04-12

**Authors:** Nawras L. Al Shabuol, Belal S. Obeidat

**Affiliations:** Department of Animal Production, Faculty of Agriculture, Jordan University of Science and Technology, Irbid 22110, Jordan

**Keywords:** Awassi lambs, faba beans, growth performance, nutrient intake and digestibility

## Abstract

**Background and Aim:**

The high price of conventional diet ingredients led livestock producers to search for alternative feed sources such as faba beans (*Vicia faba* L.; FB). This study aimed to evaluate the effect of feeding FB on the growth performance of lambs.

**Materials and Methods:**

A total of 24 male lambs were distributed randomly into two groups and fed a control diet (CON; n=12) and 200 g/kg FB (FB200; n=12) dietary dry matter (DM). The study lasted for 70 days. The first 7 days were used for acclimatization, followed by 63 days of data collection (i.e., nutrient intake and digestibility and growth performance). A complete randomized design was used for the statistical analysis.

**Results:**

The average initial body weight (BW) (20.54±0.798 kg) was similar between the diet treatment groups. Lambs fed the FB200 diet demonstrated higher (p≤0.008) nutrient intake than lambs fed the CON diet. The FB200 diet tended to improve the digestibility of DM, crude protein, and acid detergent fiber more than the CON diet (p≤0.072). Neutral detergent fiber and ether extract digestibility were higher (p<0.05) in lambs fed the FB200 diet than those fed the CON diet. Next, nitrogen retention increased (p<0.05) in lambs fed the FB200 diet compared with the CON diet. Final BW did not differ (p=0.221) between the two groups. However, the average daily gain was higher (p=0.028) in lambs fed the FB200 diet than in lambs fed the CON diet. Furthermore, the cost of gain decreased more (p=0.04) with the FB200 diet than with the CON diet.

**Conclusion:**

The results obtained in this study demonstrate the feasibility of using FB in feeding growing lambs, as it was shown to improve growth performance and reduce the cost of diet and gain.

## Introduction

An unprecedented increase in the price of feed ingredients occurred in the Jordanian market, which directly impacted livestock breeders, more so because the government reduced subsidies on some basic animal feed components. The price of red meat in the market does not compensate for the additional cost of production [1]. Furthermore, the lack of pastures in arid and semi-arid areas is challenging for livestock producers, especially sheep farmers. The high prices of feed ingredients (e.g., barley, wheat, and soybeans) forced livestock keepers and researchers to search for high-quality alternative feed materials that are less expensive [2,3].

Faba beans (*Vicia faba* L.; FB) are an important legume in human nutrition. However, when they are graded, large quantities that are unfit for human consumption exist, as well as broken and small beans that can be fed to livestock to replace protein sources, the most expensive feed. In Jordan, FB is available in large quantities from two sources, locally grown and imported from abroad. While FB is not widely used for feeding small ruminants, several scientific studies reported the use of FB in non-ruminants [4-6]. These studies concluded that the protein portion in FB can compare qualitatively to soybean protein [7]. FB legume seeds with a relatively high nutritional value are reasonably cheap and widely available in the Mediterranean region [8]. The content of starch and amino acids in FB is high [9] and well-balanced in relation to raw protein [8]. Researchers noted that diets used for fattening lambs relied heavily on FB for similar growth performance than conventional diets that rely heavily on soybean meal as a significant protein source [8]. A study reported by Lanza *et al*. [8], found that feed mixtures containing FB demonstrated a high non-protein nitrogen (N) level and immediately degraded in the rumen. Another study found that the level of cholesterol in the blood decreases with FB compared with soybean and pea groups. Beans can be used as a suitable replacement for soybean meal to fatten sheep, thus reducing cost and increasing profitability [10] effectively. The previous studies suggest that FB can be used as an alternative feedstuff to feed small ruminant animals.

We hypothesized that feeding FB to small ruminants would reduce production costs without affecting performance when used to replace soybean meal in traditional diets. This study aimed to assess the effects of FB feeding on the performance and cost of the production of growing lambs.

## Materials and Methods

### Ethical approval

The study was approvedby Jordan University of Science and Technology (JUST) Institutional Animal Care and Use Committee (#: 16/03/02/495).

### Study period and location

The study was conducted from November 2020 to January 2021 at the Agricultural Research and Training Unit/Faculty of Agriculture at JUST. Samples collected during the study were analyzed at the Department of Animal Production Laboratory.

### Animals, diets, pens, and laboratory work

In a completely randomized design, 24 lambs (body weight [BW]=20.54±0.798 kg) were separated into two groups and fed different diets. These diets were the following: (1) The control diet (CON; n=12) and (2) 200 g/kg FB (FB200; n=12) of dietary dry matter (DM). Barley grains and soybean meal were partially replaced by FB. The diets were formulated to contain 160 g/kg crude protein (CP) of dietary DM for growing lambs [11]. During the study, both diets were mixed biweekly in the farm feed mill and sampled after mixing to ensure consistency of chemical composition. The study lasted for 70 days, with the first 7 days used for adaptation and the following 63 days used to collect data. Lambs were purchased from a local farm and shipped directly to the animal farm at JUST. The health status of the lambs was assessed, and they were weighed, ear-tagged, and treated against internal parasites with 2 mL/lamb of ivermectin 1% (Ivermic, Laboratorios Microsules Uruguay S.A, Uruguay). The lambs were housed individually in shaded concrete pens (0.75×1.5 m), each equipped with plastic waterers (7 L) and feeders (10 L).

Nutrient intake was measured once a day for each lamb after subtracting feed refused from that offered. The BWs of the lambs were measured at the start of the study and every 2 weeks thereafter. Measurements were made before the morning feeding to determine average daily gain (ADG) and feed conversion ratio (DM intake; gain). All ingredients were provided *ad libitum* (110% of the previous day’s intake) to the lambs as a total mixed ration diet (Table-1). Next, freshwater was provided hourly during the study.

**Table 1:** Ingredients and chemical composition of diets containing FB fed to Awassi lambs.

Item	Diet^a^		

	CON	FB200	FB
Ingredients (g/kg DM)			
Barley grain	530	390	
Soybean meal	180	120	
FB	0	200	
Wheat straw	270	270	
Salt	10	10	
Limestone	9	9	
Vitamin-mineral premix^b^	1	1	
Feed cost/ton (US$)^c^	365	319	
Nutrients (g/kg DM)			
DM	905	908	925
Crude protein	163	164	234
Neutral detergent fiber	290.7	312.9	272
Acid detergent fiber	189.5	194.3	104
Ether extract	18.9	23.9	43

a The diets were the CON and 200 g/kg FB (FB200) of dietary DM.

b Composition per kg contained: Vitamin A, 600,000 IU; Vitamin D3, 200,000 IU; Vitamin E, 75 mg, Vitamin K3, 200 mg; Vitamin B1, 100 mg; Vitamin B5, 500 mg; lysine 0.5%; DL-methionine, 0.15%; manganese oxide, 4000 mg; ferrous sulfate, 15,000 mg; zinc oxide, 7000; magnesium oxide, 4000 mg; potassium iodide, 80 mg; sodium selenite, 150 mg; copper sulfate, 100 mg; cobalt phosphate, 50 mg; dicalcium phosphate, 10,000 mg.

c Calculated based on the prices of diet ingredients of the year 2021, FB=Faba bean, DM=Dry matter, CON=Control diet

Samples of FB, diets, and refusals were dried at 55°C for 3-4 days using a forced-air oven to reach a constant weight and then ground to pass through a 1 mm screen (Brabender OHG Duisdurg, Kulturstrase 51-55, type 880845, Nr 958084, Germany). The samples were later analyzed for DM, CP, neutral detergent fiber (NDF), acid detergent fiber (ADF), and ether extract (EE) contents using procedures of AOAC [12] and Van Soest *et al*. [13], with modifications for use in an ANKOM 2000 fiber analyzer apparatus (Ankom Technology Cooperation, Fairport, NY).

### Digestibility and N balance

On day 49 of the growing period, five lambs from each group were selected randomly and housed in separate metabolism crates (1.05×0.80 m) to assess nutrient digestibility and N balance. The animals were allowed to adapt to the metabolism crates for 5 days, followed by another 5-day period where refusals were sampled for further analysis. Afterward, the daily fecal outputs were collected, weighed, and recorded, with 10% being kept for subsequent analyses. Furthermore, urine was collected in plastic containers and then weighed and recorded with 5% stored (−20°C) to evaluate N balance. Each bottle contained 50 mL of 6N HCL to prevent ammonia loss.

Fecal samples were dried at 55°C in a forced-air oven to reach a constant weight with the air equilibrated. Then, the samples were ground to pass through a 1 mm screen and analyzed for DM, CP, NDF, ADF, and EE [12,13]. Urine samples were analyzed for CP (Kjeldahl procedure) to calculate N retention.

### Statistical analysis

Data were analyzed using the MIXED procedure of SAS (Version 8.1, 2000, SAS Inst. Inc., Cary, NC, USA). For all data, the fixed effects included only treatment, where the lamb was the random variable. The least-square means were separated using appropriate pairwise t-tests if the fixed effects were significant (p<0.05).

## Results

The inclusion of FB in the diet of growing lambs decreased the cost of the diet compared with the CON diet (Table-1). In addition, this inclusion did not intensively change the chemical composition of the diet. Nutrient intake is shown in Table-2. Lambs fed the FB200 diet showed a higher (p≤0.008) intake of DM, CP, NDF, ADF, and EE than lambs fed the CON diet.

**Table 2:** Nutrient intake of Awassi lambs fed diets containing FB.

Item	Diet^a^			

	CON (n=12)	FB200 (n=12)	SEM	p-value
Nutrient intake, g/d				
DM	1060	1127	17.53	0.008
Crude protein	173	185	2.87	0.004
Neutral detergent fiber	308	353	5.34	<0.0001
Acid detergent fiber	201	219	3.37	0.001
Ether extract	20.0	26.4	0.381	<0.0001

a The diets were the CON and 200 g/kg FB (FB200) of dietary DM. FB=Faba bean, DM=Dry matter, SEM=Standard error of the mean, CON=Control diet

The digestibility of nutrients and N balance data is shown in Table-3. The FB200 diet tended (p≤0.072) to improve the digestibility of DM, CP, and ADF more than the CON diet. The NDF and EE digestibility increased (p<0.05) in lambs fed the FB200 diet compared with lambs fed the CON diet. The diets did not differ regarding N intake (p=0.376); however, N loss in feces and urine tended to increase (p≤0.083) more in the CON diet than in the FB200 diet. In lambs fed the FB200 diet compared with lambs fed the CON diet, N retained (g/d) and N retention (%) increased (p<0.05).

**Table 3:** Effects of feeding FB on nutrient digestibility and N balance of Awassi lambs.

Item	Diet^a^			

	CON (n=5)	FB200 (n=5)	SEM	p-value
Digestibility, %				
Dry matter	71.98	77.63	1.643	0.072
Crude protein	73.64	77.64	1.142	0.069
Neutral detergent fiber	61.15	71.17	2.323	0.038
Acid detergent fiber	48.81	58.75	2.689	0.059
Ether extract	73.99	88.67	3.464	<0.05
N balance				
N intake, g/d	27.08	25.00	1.800	0.376
N in feces, g/d	6.15	4.37	0.635	0.083
N in urine, g/d	6.11	3.60	0.777	0.084
N retained, g/d	14.82	18.63	1.620	<0.05
Retention, g/100 g	54.91	74.52	3.081	<0.05

a The diets were the CON and 200 g/kg FB (FB200) of dietary DM. FB=Faba bean, DM=Dry matter, SEM=Standard error of the mean, CON=Control diet

Growth performance data are shown in Table-4. Initial and final BW was similar (p≥0.221) between the two diets. However, ADG was higher (p=0.028) for lambs fed the FB200 diet compared with lambs fed the CON diet. The cost of gain was lower (p=0.04) for the FB200 diet group than CON diet group.

**Table 4:** Growth performance of Awassi lambs fed diets containing FB.

Item	Diet^a^			

	CON (n=5)	FB200 (n=5)	SEM	p-value
Initial weight, kg	20.58	20.50	0.798	0.942
Final weight, kg	32.92	34.98	1.141	0.221
Average daily gain, g/d	195.8	229.9	12.21	0.028
Feed efficiency(DMI: ADG)^b^	5.53	5.08	0.263	0.255
Total gain, kg	12.33	14.48	0.769	0.078
Cost/kg (US$)	1.98	1.63	0.123	0.041

a The diets were the CON and 200 g/kg FB (FB200) of dietary DM.

b DMI: ADG=Dry matter intake: average daily gain, FB=Faba bean, DM=Dry matter, ADG=Average daily gain, SEM=Standard error of the mean, CON=Control diet

## Discussion

This study aimed to evaluate the effect of feeding FB on nutrient intake, digestibility, and growth performance in lambs. Therefore, this study experimented with alternative feeds such as FB in the diet of ruminants to obtain requirements for gains, reduced costs, and increased profitability. Table-1 summarizes the results of the experiment. Researchers found that by replacing a portion of soybean meal and barley grains with a FB diet at 200 g/kg DM, diet cost decreased by 13% compared to the cost of the CON diet. This cost reduction is due to the fact that legume seeds, such as broken and heterogeneous FB grains, are not consumed by humans, making them readily available for feeding ruminants, especially lambs. Our results agree with a previous study on Awassi lambs, which showed a reduction of 13.5% in the cost of alternative feed by replacing barley grains with FB. The use of FB that is subpar for human consumption as an alternative animal diet demonstrates the potential to reduce and stabilize fluctuations in the cost of grain and other feed sources [14].

The CP and DM contents of both the CON diet and FB200 diet were comparable, except for the ADF, NDF, and EE contents, which were higher in the FB200 diet than in the CON diet. These differences were because the ADF, NDF, and EE contents of FB were higher than that of soybean meal and barley grains (whole). These results are similar to those of other studies [15-17].

Our results are consistent with Hartwell *et al*. [14] and Bonanno *et al*. [18], who showed that using a diet containing FB increased the intake of DM and CP in lambs more than a diet containing soybean meal as a source of protein. Furthermore, Purroy *et al*. [15] reported that the intake of DM and CP in lambs is higher when they are on a FB diet than on any other source of protein diet from legumes. The cause of higher intake in lambs on a FB diet could be because lambs select feeds based on digestibility, palatability, and flavor [19]. It could also be due to the presence of more antinutritional factors in soybean meal diets than in FB diets [20]. In addition, the level and activity of antinutritional factors, such as tannins, in FB exhibit a more negligible effect on ruminants [15]. These results are in line with a previous study by Bonanno *et al*. [18] who reported that the presence of antinutritional factors did not negatively affect the palatability of FB.

The EE intake for lambs was also higher in the FB200 diet than in the CON diet, possibly due to the high EE present in the FB200 diet, which also applies to ADF and NDF intake, which were higher in the FB200 diet than in the CON diet. In a previous study, chemical composition evaluation showed that a diet containing FB at 200 g/kg was higher in ADF (0.16 vs. 0.21 g/100 g DM) and NDF (0.38 vs. 4.6 g/100 g DM) than a diet containing soybean meal [16]. In another study, the chemical composition of a FB diet at 300 g/kg was shown to contain high EE (32 g/kg DM) [21].

Furthermore, the results presented in Table-3 show improved digestibility with the FB200 diet compared with the CON diet. The expectation exists that the increase in the digestibility of NDF, ADF, and EE is due to their high percentage in the FB200 diet, as mentioned previously. In addition, the greater improvement in the digestibility of DM, CP, NDF, and ADF in the FB200 diet compared with the CON diet and the difference in the N balance ratios in both diets may be due to antinutritional factors, enzymatic CP degradation, soluble N, DM, and protein rumen degradation. A study conducted by Masoero *et al*. [17] on the chemical analysis of FB and soybean meals which showed that compared to soybean meals, FB meals contain lesser amounts of antimicrobials such as genistein (0 vs. 0.70 ppm), daidzein (0.10 vs. 1.60 ppm), and antitrypsin (0.78 vs. 1.30 mg/g) treatment activity. The results also showed that FB contains enzymatic CP degradation and soluble N (81.76 and 71.12, respectively), which were higher in FB meals compared to soybean meals (76.18 and 12.71, respectively) (data in g/100 g of initial N). The difference in the chemical composition of FB and soybean meals also influences feed intake and N balance. The same experiment by Masoero *et al*. [17] showed differences in *in vitro* DM and protein rumen degradation (g/100 g). After 8 and 24 h of incubation, the percentage of rumen degradation was higher in FB meals than in soybean meals, for both DM and CP.

Furthermore, Table-4 shows the growth performance of Awassi lambs and an improvement in the ADG in the FB200 diet group compared to the CON diet group. It also shows the absence of significant differences in the other measures. These results are in agreement with a previous study by Boukhris *et al*. [16], which showed that no significant differences were found in the final weight (kg) and daily gain (g/day) between a group of Awassi lambs fed FB at 200 g/kg and a group fed soybean meal. Furthermore, a study conducted by Hartwell *et al*. [14] on Awassi lambs showed no effect on total weight gain, final weight, and ADG when barley grain was replaced with FB in the diet. In another study by Purroy *et al*. [15], who compared a diet containing soybean meal and another containing FB with the same level of protein in the diet; researchers found that ADG was higher in the FB diet than in the soybean meal diet, without affecting the final weight.

Contrary to this, Facciolongo *et al*. [10] conducted a study on the substitution of soybean meal with FB and other legumes such as lupin and peas; the results recorded a higher slaughter weight in the FB diet group (23.07 kg) compared to the soybean meal diet group (19.93 kg) due to the difference in ADG between the FB diet group and soybean meal diet group (0.21 and 0.16 kg/d, respectively). Furthermore, the experiment results are in line with the results of an experiment conducted by Bonanno *et al*. [18] on the use of FB as an alternative protein source to soybeans. These results showed no significant differences in the final weight and ADG in both diets due to similarities in the daily feed intake and feed conversion ratio.

The cost of gain decreased with the FB200 diet compared to the CON diet, which may be due to the lower price of FB compared with the cost of barley grains and soybean meal during the study period. Many studies dealing with alternative feeds from plant or agro-industrial by-products, such as sweet lupin [21,22], olive cake [23], black cumin meal [3], and olive cake and *Atriplex halimus* L. [2], obtained similar results by providing diets that were more economically feasible and beneficial to livestock production.

## Conclusion

The present study results showed that the inclusion of FB at 200 g/kg improved digestibility, feed intake, and growth performance of lambs. In addition, the results showed a beneficial economic effect through reduced production costs associated with the FB diet compared with the CON. Further studies are needed to study the effects of feeding FB at different levels and species.

## Authors’ Contributions

BSO and NLA: Designed and conducted the study, data analysis, and drafted and revised the manuscript. Both authors read and approved the final manuscript.
